# Performance of 2 Single-Item Screening Questions to Identify Future Homelessness Among Emergency Department Patients

**DOI:** 10.1001/jamanetworkopen.2022.26691

**Published:** 2022-08-15

**Authors:** Thomas Byrne, Mindy Hoang, Ann Elizabeth Montgomery, Eileen Johns, Marybeth Shinn, Tod Mijanovich, Dennis Culhane, Kelly M. Doran

**Affiliations:** 1 School of Social Work, Boston University, Boston, Massachusetts; 2 University of Cincinnati College of Medicine, Cincinnati, Ohio; 3 School of Public Health, University of Alabama at Birmingham, Birmingham; 4 Birmingham Veterans Affairs Health Care System, Birmingham, Alabama; 5 New York City Center for Innovation through Data Intelligence, New York, New York; 6 Department of Human and Organizational Development, Peabody College, Vanderbilt University, Nashville, Tennessee; 7 Department of Applied Statistics, Social Sciences, and Humanities, New York University Steinhardt School, New York, New York; 8 School of Social Policy and Practice, University of Pennsylvania, Philadelphia; 9 Department of Emergency Medicine, New York University School of Medicine, New York, New York; 10 Department of Population Health, New York University School of Medicine, New York, New York

## Abstract

**Importance:**

Despite increasing interest in assessing patient social needs in health care settings, there has been little research examining the performance of housing-related screening questions.

**Objective:**

To examine the performance of 2 single-item screening questions assessing emergency department (ED) patients’ self-perceived risk of future homelessness.

**Design, Setting, and Participants:**

This prospective cohort study was conducted among a randomly selected sample of adult ED patients from 2016 to 2017 in a public hospital ED in New York City. Data were analyzed from September 2019 through October 2021.

**Exposures:**

Responses on patient surveys conducted at the baseline ED visit for 2 single-item screening questions on self-perceived risk for future housing instability and homelessness were collected. One question asked patients if they were worried about having stable housing in the next 2 months, and the other question asked them to rate the likelihood that they would enter a homeless shelter in the next 6 months.

**Outcomes:**

Homeless shelter entry 2, 6, and 12 months after an ED visit, assessed using shelter administrative data in the study city, which was linked with participant baseline survey responses.

**Results:**

There were 1919 study participants (976 [51.0%] men and 931 [48.6%] women among 1915 individuals with gender data; 700 individuals aged 31-50 years [36.5%] among 1918 individuals with age data; 1126 Hispanic or Latinx individuals [59.0%], 368 non-Hispanic Black individuals [19.3%], and 225 non-Hispanic White individuals [11.8%] among 1908 individuals with race and ethnicity data). Within 2, 6, and 12 months of the ED visit, 45 patients (2.3%), 66 patients (3.4%), and 95 patients (5.0%) had entered shelter, respectively. For both single-item screening questions, participants who answered affirmatively had significantly higher likelihood of future shelter entry at each time point examined (eg, at 2 months: 31 participants responding yes [6.5%] vs 14 participants responding no [1.0%] to the question concerning being worried about having stable housing in the next 2 months). Sensitivity of the screening questions ranged from 0.27 to 0.69, specificity from 0.76 to 0.97, positive predictive value from 0.07 to 0.27, and area under the receiver operating characteristic curve from 0.62 to 0.72.

**Conclusions and Relevance:**

This study found that 2 single-item screening questions assessing ED patient self-perceived risk of future housing instability and homelessness had adequate to good performance in identifying risk for future shelter entry. Such single-item screening questions should be further tested before broad adoption.

## Key Points

**Question:**

Can single-item screening questions accurately identify risk for future homelessness among emergency department patients?

**Findings:**

In this prospective cohort study of 1919 emergency department patients, 3.4% who were not homeless at baseline entered a homeless shelter in the next 6 months. Single-item screening questions capturing self-rated risk for homelessness had sensitivities ranging from 0.27 to 0.69 and specificities ranging from 0.76 to 0.97 for identifying shelter entry in the next 6 months.

**Meaning:**

These findings suggest that single-item screening questions may hold promise for identifying emergency department patients at risk for future homelessness who may benefit from referrals to homelessness prevention services.

## Teaser

This cohort study investigates the performance of single-item screening questions in identifying future homelessness among patients in the emergency department.

## Introduction

The association of homelessness^1,2^ and housing insecurity^3^ with adverse health outcomes and higher health care costs has prompted increasing interest in screening for homelessness and housing insecurity in health care settings. A number of screening tools use self-reported information collected in clinical settings to identify homelessness or other forms of housing insecurity.^4,5,6,7,8^ Such tools may be particularly valuable for use in emergency departments (EDs) given evidence of increased rates of housing insecurity among ED patients,^9,10,11^ some of whom may not be using other forms of health care or may be experiencing health issues that could contribute to onset of homelessness. However, prior research on screening tools for housing instability have focused largely on their administration in outpatient^4,5,12,13,14^ or pediatric settings,^6,15,16,17,18,19^ with limited attention to the ED setting.

Targeting services to prevent the onset of an episode of homelessness among patients presenting for ED care may be particularly important. Our research team developed an empirically based homelessness risk screening tool using patient-collected data from a New York City (NYC) ED, linked with data from the city’s emergency shelter system.^20^ The screening tool identified as having the best performance in identifying future shelter entry comprised 3 questions, 2 of which asked about recent use of or application for shelter services. Such screening questions may be less relevant in settings where unsheltered homelessness is more prevalent than in NYC, which has a legal right to shelter^21^ and predominance of sheltered homelessness. Furthermore, reliance on prior shelter use to estimate risk for future shelter entry is of little value if the objective is to prevent first-time homelessness.

In this study, we evaluated the ability of 2 distinct single-item screening questions administered to ED patients to identify risk for future homelessness. A single-item question eliciting information on patient risk for future homelessness may be particularly valuable given that screening tools that are brief and easy to administer (ideally, single questions) may be implemented with greater frequency and fidelity, particularly in busy ED settings.^4^ Furthermore, we aimed to examine how screening questions that asked patients themselves to forecast their risk performed compared with screening tools based on past events or behavior (eg, past shelter use).

## Methods

The Institutional Review Board at New York University Grossman School of Medicine approved this cohort study. Written informed consent was obtained from all participants. We followed the Strengthening the Reporting of Observational Studies in Epidemiology (STROBE) reporting guideline.

### Study Design and Setting

We conducted a prospective cohort study of a randomly selected sample of ED patients. These patients completed an in-person survey and were followed up prospectively using administrative databases that tracked shelter use to assess their subsequent homelessness.

### Study Setting and Population

We recruited patients who presented to a public hospital ED in NYC November 2016 to September 2017. Patients were eligible if they were aged 18 years or older and spoke English or Spanish. Patients were ineligible if they were medically unstable, in psychological distress, in police or prison custody, could not provide informed consent (eg, had dementia or were too intoxicated to participate), did not live in NYC, or had already participated. We used a random sampling scheme to approach patients, which we have described previously.^22^

### Measures

Research assistants verbally administered a 20- to 40-minute survey to participants during ED visits. Survey responses were captured in Research Electronic Data Capture (REDCap) software using Wi-Fi–enabled tablet computers (iPads, Apple).

Surveys included 2 single-item screening questions for self-perceived risk of future homelessness: (1) “Are you worried or concerned that in the next 2 months you may not have stable housing that you own, rent, or stay in as part of a household?” with responses of “yes” or “no,” and (2) “How likely do you think it would be that you would have to use a homeless shelter in the next 6 months?” with 4 response options ranging from “very unlikely” to “very likely.” The first question was taken from the US Department of Veterans Affairs Homelessness Screening Clinical Reminder (HSCR), a screening instrument administered in outpatient settings throughout the Veterans Health Administration.^5^ The HSCR has good reliability and has been validated against a measure of current housing status^23^ but not future homelessness. The second question was developed by the study team in consultation with a stakeholder advisory board.

Participants were asked about race and ethnicity in 2 separate questions, and they self-identified for both. Race and ethnicity were assessed because of known racial and ethnic disparities in US homelessness, which are related to structural factors including long-standing systemic racism in access to housing opportunities. Response options for race were American Indian or Alaska Native, Black, Native Hawaiian or other Pacific Islander, other Asian, Southeast Asian or from the Indian Subcontinent, White, more than 1 race, and other race. For ethnicity, participants could respond yes or no to being Hispanic or Latinx. Race and ethnicity categories were combined for analyses as Hispanic or Latinx, non-Hispanic Black, non-Hispanic White, and non-Hispanic other. Investigators combined categories (using simple bivariate cross-tabulation) to more accurately reflect how participants in this study identified themselves. Namely, most study participants identified as Hispanic or Latinx, and when research assistants asked participants the separate question about race, they often said that they did not identify as any particular race (ie, they self-identified as Hispanic or Latinx only), resulting in a large number of “other” responses.

Participants provided identifying information (ie, name, date of birth, and Social Security number if they had one) and gave consent for linkage of their survey results with shelter records from the NYC Department of Homeless Services Client Assistance and Rehousing Enterprise System (CARES) database. CARES captures more than 90% of shelter stays in NYC and includes dates of shelter entry and exit. Data linkage with CARES was performed by the NYC Center for Innovation Through Data Intelligence using deterministic and probabilistic matching procedures described previously.^22^

### Statistical Analysis

We used simple descriptive statistics to describe study participant characteristics, responses to single-item screening questions, and incidence of shelter entry (based on CARES data) at different times after survey completion. We used 2-sided χ^2^ or Fisher exact tests as appropriate to conduct bivariate tests of associations between shelter entry and demographic characteristics, drug or unhealthy alcohol use in the prior year, and responses to single-item screening questions for self-perceived risk of future homelessness. Additionally, we examined the performance of single-item screening questions in identifying future shelter entry, examining sensitivity, specificity, positive predictive value (PPV), and area under the receiver operating characteristic curve (AUROC), which was calculated using univariable logistic regression models. All bivariate tests were conducted using R statistical software version 4.1.0 (R Project for Statistical Computing), and univariable logistic regression models were estimated using SAS statistical software version 9.0 (SAS Institute).

Given that our interest was in screening for future homelessness, we excluded from our analytic sample participants who self-reported that they were currently homeless (ie, spent the past night in a shelter or unsheltered) or who were documented in CARES as having used a shelter in the past week. For the question asking participants to rate their likelihood of using a shelter in the next 6 months, we conducted 2 analyses: first, dichotomizing responses to include only individuals who reported that this was “very likely” as a positive screen and then again including those who reported that this was “very likely” or “somewhat likely.”

We conducted 2 secondary subgroup analyses. Given documented associations of substance use and homelessness among ED patients,^24^ we conducted a subgroup analysis among participants who reported drug use or unhealthy alcohol use in the past year. Second, given our interest in whether screening questions could identify first-time homelessness, we repeated our analysis for the subgroup of participants with no prior record of shelter use in CARES. Data were analyzed from September 2019 through October 2021.

## Results

Of 6097 patients assessed for eligibility, 3173 patients (52.0%) were ineligible, most frequently because they were too ill (858 patients), were intoxicated (496 patients), or did not speak English or Spanish (489 patients). Among 2924 eligible patients, 2396 individuals (81.9%) participated. We removed duplicate records from 84 patients who participated more than once and 3 patients who did not give identifying information, leaving 2309 participants. Of these, we excluded 380 individuals (16.5%) who were currently homeless and an additional 10 individuals (0.4%) who did not respond to the single-item screening questions, resulting in a final analytic sample of 1919 participants.

These study participants (976 [51.0%] men, 931 [48.6%] women, and 8 transgender individuals [0.4%] among 1915 individuals with gender data) represented a range of ages (443 individuals aged 18-30 years [23.1%], 700 individuals aged 31-50 years [36.5%], 529 individuals aged 51-65 years [27.6%], and 246 individuals aged >65 years [12.8%] among 1918 individuals with age data). Among 1908 participants with race and ethnicity data, 1126 individuals self-identified as Hispanic or Latinx (59.0%), 368 individuals as non-Hispanic Black (19.3%), 225 individuals as non-Hispanic White (11.8%), and 189 individuals as other non-Hispanic race (9.9%) (Table 1).^25,26^ CARES data indicated that 45 participants (2.3%), 66 participants (3.4%), and 95 participants (5.0%) in our analytic sample used a NYC shelter within 2, 6, and 12 months of their ED visit, respectively. Shelter use was significantly higher for men, non-Hispanic Black participants, and those who screened positive for unhealthy alcohol or any drug use.

**Table 1.** Participant Characteristics and Summary of Homeless Shelter Use

Characteristic	Overall, No. (%) (N = 1919)	Entered shelter after ED visit, No. (%)					
		2 mo (n = 45 [2.3%])	*P* value	6 mo (n = 66 [3.4%])	*P* value	12 mo (n = 95 [5.0%])	*P* value
Gender (n = 1915)							
Men	976 (51.0)	36 (3.7)	<.001	55 (5.6)	<.001	79 (8.1)	<.001
Women	931 (48.6)	8 (0.9)		9 (1.0)		14 (1.5)	
Transgender	8 (0.4)	1 (12.5)		1 (12.5)		1 (12.5)	
Age, y (n = 1918)							
18-30	443 (23.1)	8 (1.8)	.41	11 (2.5)	.15	13 (2.9)	.01
31-50	700 (36.5)	19 (2.7)		27 (3.9)		35 (5.0)	
51-65	529 (27.6)	15 (2.8)		23 (4.4)		38 (7.2)	
>65	246 (12.8)	3 (1.2)		4 (1.6)		8 (3.3)	
Race and ethnicity (n = 1908)^a^							
Hispanic or Latinx	1126 (59.0)	14 (1.2)	<.001	20 (1.8)	<.001	27 (2.4)	<.001
Non-Hispanic Black	368 (19.3)	25 (6.8)		33 (9.0)		49 (13.3)	
Non-Hispanic White	225 (11.8)	4 (0.2)		9 (4.0)		10 (4.4)	
Non-Hispanic other^b^	189 (9.9)	2 (1.1)		3 (1.6)		8 (4.2)	
Unhealthy alcohol or any drug use in past year (n = 1916)^c^							
Yes	701 (36.6)	32 (4.6)	<.001	47 (6.7)	<.001	71 (10.1)	<.001
No	1215 (63.4)	13 (1.1)		18 (1.5)		23 (1.9)	

^a^ Participants were asked about race and ethnicity in 2 separate questions, and they self-identified for both.

^b^ Other includes participants who identified as non-Hispanic and American Indian or Alaska Native, Native Hawaiian or other Pacific Islander, other Asian, Southeast Asian or from the Indian Subcontinent, more than 1 race, or other.

^c^ Validated single-item screening questions were used for any unhealthy alcohol use (defined as ≥4 drinks in a day for women and ≥5 drinks in a day for men) in the past year and any drug use (including marijuana) in the past year.^25,26^

One-quarter of participants (479 individuals [25.0%]) reported that they were worried about having stable housing in the next 2 months. Fewer participants said they were very likely (80 individuals [4.2%]) or somewhat likely (112 individuals [5.9%]) to use a homeless shelter in the next 6 months among 1910 participants answering this question. Table 2 shows rates of subsequent shelter use by responses on single-item screening questions. Rates of shelter use among individuals who responded yes that they were worried they may not have stable housing in the next 2 months were significantly higher than among those who responded no at 2 months (31 participants [6.5%] vs 14 participants [1.0%]), 6 months (43 participants [9.0%] vs 23 participants [1.6%]), and 12 months (57 participants [11.9%] vs 38 participants [2.6%]) after the ED visit. The same pattern held true for the screening question on participant self-rated likelihood of future shelter use.

**Table 2.** Comparison of Responses to Single-Item Screening Questions

Screening question	Overall, No. (%) (N = 1919)	Entered shelter after ED visit, No. (%)					
		2 mo	*P* value	6 mo	*P* value	12 mo	*P* value
Worried about housing in next 2 mo							
Yes	479 (25.0)	31 (6.5)	<.001	43 (9.0)	<.001	57 (11.9)	<.001
No	1440 (75.0)	14 (1.0)		23 (1.6)		38 (2.6)	
How likely are you to enter a shelter in the next 6 mo (n = 1910)							
Very likely	80 (4.2)	13 (16.3)	<.001	18 (22.5)	<.001	26 (32.5)	<.001
Somewhat likely	112 (5.9)	10 (8.9)		13 (11.6)		13 (11.6)	
Somewhat unlikely	145 (7.6)	7 (4.8)		10 (6.9)		12 (8.3)	
Very unlikely	1573 (82.3)	15 (1.0)		25 (1.6)		43 (2.7)	

Table 3 summarizes sensitivity, specificity, PPV, and AUROC for each single-item screening question. The AUROC value (0.72) for the model identifying shelter use within 2 months of survey completion based on whether participants were worried about their housing in the next 2 months was considered acceptable by conventional guidelines.^27^ The AUROC was similar when identifying shelter use within 6 months (0.71) and 12 months (0.68). Sensitivity was 0.69 in identifying shelter entry at 2 months, decreasing to 0.60 for the 12-month outcome. Specificity was similar (ranging from 0.76 to 0.77) regardless of follow-up period. PPV for identifying shelter use within 2 months of survey completion was 0.07, increasing to 0.12 by 12 months (ie, 12% of participants responding affirmatively had used a shelter). The screening question response of very likely for self-judged likelihood of entering a shelter in the next 6 months had higher specificity (range, 0.96-0.97) but lower sensitivity (range, 0.27-0.29) and AUROC (range, 0.62-0.63) in identifying future shelter entry compared with the question on worry about stable housing in the next 2 months. When adding responses of somewhat likely to the definition of a positive screen, sensitivity improved (range, 0.41-0.51) and specificity remained high (0.91), but PPV decreased from 0.27 to 0.20 at 6 months.

**Table 3.** Performance of Single-Item Screening Questions in Identifying Future Homeless Shelter Use

Screening question	Shelter entry after ED Visit											
	2 mo				6 mo				12 mo			
	Sensitivity	Specificity	PPV	AUROC	Sensitivity	Specificity	PPV	AUROC	Sensitivity	Specificity	PPV	AUROC
Worried about housing in next 2 mo	0.69	0.76	0.07	0.72	0.65	0.76	0.09	0.71	0.60	0.77	0.12	0.68
How likely are you to enter shelter in the next 6 mo?												
Very likely	0.29	0.96	0.16	0.63	0.27	0.97	0.22	0.62	0.27	0.97	0.27	0.62
Very or somewhat likely	0.51	0.91	0.12	0.71	0.47	0.91	0.16	0.69	0.41	0.91	0.20	0.66

Abbreviations: AUROC, area under the receiver operating characteristic curve; PPV, positive predictive value.

Screening question performance for the subgroup of participants with drug or unhealthy alcohol use was similar to that observed for the full sample, with higher PPVs as expected given the larger proportion of individuals who entered a shelter (eTable 1 in the Supplement). For the subgroup of participants with no prior record of shelter use in CARES, screening questions had similar performance to that for the full population, with lower PPVs as expected given the lower prevalence of postbaseline shelter use (eTable 2 in the Supplement).

## Discussion

To our knowledge, this cohort study is the first study to assess how well screening questions capturing self-perceived risk of future homelessness can identify onset of a future episode of homelessness. Our findings are important in the context of increasing interest in screening for and intervening to address social needs in EDs.^28^ Nearly 40% of New England EDs reported screening for at least 1 health-related social need,^29^ yet there is a dearth of evidence about the validity of commonly used screening questions. The potential need is significant: our finding that 25% of patients were worried that they may not have stable housing in the next 2 months is similar to the findings from a study conducted in an urban ED in Oakland, California.^30^

Differences in performance between 2 screening questions examined in this study are consistent with past research showing that various housing-related screening questions used in health care settings were not necessarily interchangeable.^31^ In a prior study,^20^ our research group examined the performance of a broad array of variables in identifying future homelessness and found that variables related to patient past homelessness had higher PPVs than other individual-level variables, such as those related to medical issues, behavioral health, and other social factors. We found that a 3-item screening tool that included questions about shelter use in the past 12 months, application for shelter in the past 3 months, and lifetime history of incarceration had a sensitivity of 0.83 and PPV of 0.20 in identifying shelter entry within 6 months, metrics that suggest stronger performance than the single-item questions we examined in this study. Individual questions examined in that study about patient homelessness in the past 12 months had a higher PPV for future shelter entry than self-perception questions examined in this study but lower sensitivity than the question of whether individuals were worried about having stable housing in the next 2 months.

Ultimately, preference for which question or questions should be used will vary based on goals of screening, likelihood that individuals administering the screening will do so with fidelity, and interventions or services that a positive screen may trigger. Depending on these goals, the relatively lower performance of the single-item measures of self-reported risk for future homelessness may be acceptable given their ease of application and potential to identify risk for first-time homelessness. Decisions about which measure or measures of self-reported risk of future homelessness to use should also be based on follow-up actions or interventions targeted to patients who screen positive. For example, given its relatively high specificity, the question that asked patients to rate their likelihood of homeless shelter use may be a more appropriate tool for ruling in future homelessness, given that a more specific question may have fewer false positives (ie, a positive response to the question is more likely to be a true positive). Such a question may prompt interventions, such as housing vouchers, that are higher in cost and thus elicit concerns about efficiently targeting resources. However, for interventions that are lower cost, such as a brief consultation with a social worker or referral to homelessness prevention services in the community, the question asking patients about whether they were worried about having stable housing in the next 2 months may be more appropriate given its higher sensitivity, even if that comes at the cost of lower specificity and hence more false positives. Such a tradeoff may also be preferred for screening questions if the outcome is considered so bad that casting a wider net to catch all individuals potentially at risk is warranted. Prioritizing sensitivity over specificity and PPV is consistent with how emergency clinicians approach commonly used clinical screening tools to identify rare yet significant events.^32^

Screening questions examined in this study attempted to elicit what O’Flaherty and colleagues have called private information. This is information that is complex or idiosyncratic and fully knowable only to a given individual, which stands in contrast to public information, which is easier to collect via questionnaires (eg, have you stayed in shelter in the past year?) or can be verified through other means, such as shelter records. Using data from Australia, O’Flaherty and colleagues^33^ found that private information performed better in identifying future homelessness than public information, although the authors did not have access to what they considered the ideal measure: a single question asking about an individual’s self-perceived risk of future homelessness. Our study fills this gap, but the 2 private information questions we examined did not perform better than previously examined public information on past homelessness in our sample.^20^ Our findings highlight the inherent challenges in identifying future homelessness, including for patients themselves.

While our study makes inroads toward identifying effective social needs screening questions, it does not address the issue of real-world implementation of screening questions into ED workflows or of providing effective interventions. One 2021 review^34^ of ED interventions to address social needs found that 3% specifically addressed housing or homelessness. Experience from the Veterans Health Administration (VHA) may be instructive: 1 single-item question we examined has been in wide use for nearly a decade in the VHA,^5^ and evidence suggests that veterans who screen positive for housing instability and access VHA-provided homeless services have a decreased risk of mortality.^35^ Of course, the VHA is unique in that it has a robust network of housing interventions available for unstably housed veterans. Other effective community services to prevent homelessness exist and have been described previously.^36^ Although more work is needed to bring such programs to scale, the primary task for EDs may be identifying patients at increased risk and providing effective referrals to services.

Finally, while this was not the primary focus of our analysis, we found disparities in future shelter use among ED patients by gender, race, and ethnicity. Findings related to gender may have been amplified because the study hospital is located next to the intake shelter for men in NYC. Racial disparities in homelessness have been documented in prior research^37,38,39,40^ and bear mentioning given that they highlight that homelessness is largely a product of social and structural factors, such as structural racism resulting in unequal access to housing and employment opportunities. Regardless of patient responses to screening questions, our findings reinforce that certain groups have systematically increased risks of homelessness.

### Limitations

This study has several limitations. The study was conducted in NYC, which has a unique homelessness context. Owing to NYC’s legal right to shelter,^21^ approximately 95% of people who experience homelessness use shelters rather than staying exclusively in unsheltered locations.^41^ Therefore, we designed 1 screening question to ask about self-rated risk of future shelter use given that this was the outcome of interest for our government and community partners; this question may be less relevant in other localities. Relatedly, we relied solely on data capturing shelter use in assessing screening question performance. Owing to the relatively smaller size of the unsheltered homeless population in NYC, the impact on our study’s findings may be modest, given that shelter use is a decent proxy for literal homelessness in our setting. In other contexts, the screening question on self-rated likelihood of future shelter entry may potentially be modified to ask about self-rated likelihood of becoming homeless (not specific to shelter entry), but both screening questions should be tested in other settings. While not a limitation per se, research staff administered the screening questions as part of a study. A logical next step would be an implementation study in which screening questions are incorporated into real-world ED workflows; important questions remain about who should ask such questions and when, how information should be documented, and whether there may be unintended consequences.

## Conclusions

This cohort study contributes to the literature by evaluating the ability of 2 single-item screening questions to identify risk for future homelessness among ED patients. Future research should address implementation of such screening questions, including assessing performance in real-world implementation, as well as how to match implementation of screening questions with processes to deliver effective interventions to individuals identified as at risk of future homelessness.

## Notes

Supplement. **eTable 1.** Performance of Single-Item Screening Questions in Identifying Future Homeless Shelter Use Among 701 Participants Who Screened Positive for Unhealthy Alcohol or Any Drug Use in Prior Year **eTable 2.** Performance of Single-Item Screening Questions in Identifying Future Homeless Shelter Use Among 1839 Participants With No Prior Record of Emergency Shelter Use
