# Supplementary material for: Performance of 2 Single-Item Screening Questions to Identify Future Homelessness Among Emergency Department Patients
Source: JAMA Netw Open. 2022 Aug 15;5(8):e2226691. doi: 10.1001/jamanetworkopen.2022.26691 (PMC9379745; doi:10.1001/jamanetworkopen.2022.26691)
Supplement: Supplement. — eTable 1. Performance of Single-Item Screening Questions in Identifying Future Homeless Shelter Use Among 701 Participants Who Screened Positive for Unhealthy Alcohol or Any Drug Use in Prior Year eTable 2. Performance of Single-Item Screening Questions in Identifying Future Homeless Shelter Use Among 1839 Participants With No Prior Record of Emergency Shelter Use [file jamanetwopen-e2226691-s001.pdf]

Supplemental Online Content

Byrne T, Hoang M, Montgomery AE, et al. Performance of 2 single-item screening questions to identify future homelessness among emergency department patients. *JAMA Netw Open*. 2022;5(8):e2226691. doi:10.1001/jamanetworkopen.2022.26691

eTable 1. Performance of Single-Item Screening Questions in Identifying Future Homeless Shelter Use Among 701 Participants Who Screened Positive for Unhealthy Alcohol or Any Drug Use in Prior Year

eTable 2. Performance of Single-Item Screening Questions in Identifying Future Homeless Shelter Use Among 1839 Participants With No Prior Record of Emergency Shelter Use

This supplemental material has been provided by the authors to give readers additional information about their work.

© 2022 Byrne T et al. *JAMA Network Open.*

eTable 1. Performance of Single-Item Screening Questions in Identifying Future Homeless Shelter Use Among 701 Participants Who Screened Positive for Unhealthy Alcohol or Any Drug Use in Prior Year

	**Shelter Entry Post-ED Visit**											
	**2-months**				**6-months**				**12-months**			
	Sens.	Spec.	PPV	AUROC	Sens.	Spec.	PPV	AUROC	Sens.	Spec.	PPV	AUROC
Worried about housing in next two months	0.65	0.71	0.10	0.68	0.57	0.71	0.13	0.64	0.55	0.72	0.18	0.64
How likely are you to enter shelter?												
Very likely	0.34	0.94	0.23	0.64	0.28	0.94	0.27	0.61	0.27	0.95	0.40	0.61
Very or somewhat likely	0.63	0.87	0.19	0.75	0.53	0.87	0.23	0.70	0.44	0.88	0.29	0.66

Note: Sens.= Sensitivity; Spec.= Specificity; PPV = positive predictive value; AUROC = area under the receiver operating characteristic curve

© 2022 Byrne T et al. *JAMA Network Open.*

eTable 2. Performance of Single-Item Screening Questions in Identifying Future Homeless Shelter Use Among 1839 Participants With No Prior Record of Emergency Shelter Use

	**Shelter Entry Post-ED Visit**											
	**2-months**				**6-months**				**12-months**			
	Sens.	Spec.	PPV	AUROC	Sens.	Spec.	PPV	AUROC	Sens.	Spec.	PPV	AUROC
Worried about housing in next two months	0.65	0.76	0.02	0.71	0.69	0.77	0.05	0.73	0.67	0.77	0.06	0.72
How likely are you to enter shelter?												
Very likely	0.29	0.97	0.08	0.63	0.31	0.97	0.15	0.64	0.24	0.97	0.16	0.60
Very or somewhat likely	0.53	0.92	0.06	0.72	0.55	0.92	0.10	0.74	0.40	0.92	0.10	0.66

Note: Sens.= Sensitivity; Spec.= Specificity; PPV = positive predictive value; AUROC = area under the receiver operating characteristic curve

© 2022 Byrne T et al. *JAMA Network Open.*
